# Site-directed mutagenesis identified the key active site residues of 2,3-oxidosqualene cyclase HcOSC6 responsible for cucurbitacins biosynthesis in *Hemsleya chinensis*

**DOI:** 10.3389/fpls.2023.1138893

**Published:** 2023-03-28

**Authors:** Xia Li, Geng Chen, Qing-Qing Gao, Chun-Fan Xiang, Cheng-Xiao Yuan, Xiao-Ning Li, Yan-Yu Shu, Guang-Hui Zhang, Yan-Li Liang, Sheng-Chao Yang, Chen-Xi Zhai, Yan Zhao

**Affiliations:** ^1^ Key Laboratory of Medicinal Plant Biology of Yunnan Province, National and Local Joint Engineering Research Center on Germplasms Innovation and Utilization of Chinese Medicinal Materials in Southwest China, Yunnan Agricultural University, Kunming, China; ^2^ College of Agronomy and Biotechnology, Yunnan Agricultural University, Kunming, China; ^3^ Sibley School of Mechanical and Aerospace Engineering, Cornell University, Ithaca, NY, United States

**Keywords:** HcOSC6, cucurbitacins, molecular docking, site-directed mutagenesis, *Hemsleya chinensis*

## Abstract

*Hemsleya chinensis* is a Chinese traditional medicinal plant, containing cucurbitacin IIa (CuIIa) and cucurbitacin IIb (CuIIb), both of which have a wide range of pharmacological effects, including antiallergic, anti-inflammatory, and anticancer properties. However, few studies have been explored on the key enzymes that are involved in cucurbitacins biosynthesis in *H. chinensis*. Oxidosqualene cyclase (OSC) is a vital enzyme for cyclizing 2,3-oxidosqualene and its analogues. Here, a gene encoding the oxidosqualene cyclase of *H. chinensis* (*HcOSC6*), catalyzing to produce cucurbitadienol, was used as a template of mutagenesis. With the assistance of AlphaFold2 and molecular docking, we have proposed for the first time to our knowledge the 3D structure of HcOSC6 and its binding features to 2,3-oxidosqualene. Mutagenesis experiments on HcOSC6 generated seventeen different single-point mutants, showing that single-residue changes could affect its activity. Three key amino acid residues of HcOSC6, E246, M261 and D490, were identified as a prominent role in controlling cyclization ability. Our findings not only comprehensively characterize three key residues that are potentially useful for producing cucurbitacins, but also provide insights into the significant role they could play in metabolic engineering.

## Introduction

Cucurbitacin is a bitter triterpene found mainly in Cucurbitaceae plants, which comprise major vegetable crops used for food, feed, and medicine (Zhou et al., 2016). Cucurbitaceae plants have been used for nutritional, medicinal, and ethnoveterinary purposes. Additionally, some Cucurbitaceae fruits can be used in the cosmetic industry (Mukherjee et al., 2022). Plants that contain cucurbitacins produce a diverse range of pharmacological properties, including anti-biotics, anti-diabetes, anti-inflammatory, anti-cancerous, and antiviral properties, and associated with treatment of human diseases (Toker et al., 2003; Liu et al., 2010; Zaini et al., 2011; Lin et al., 2012; Shang et al., 2014; Zhao et al., 2014; Hassan et al., 2017; Attar and Ghane, 2019; Hua et al., 2019). The biosynthesis of cucurbitacins is also responsed to abiotic stress, such as salt, heat, and drought, suggesting an opportunity to improve their genetics for breeding and developing highly tolerant varieties (Mashela and Pofu, 2017; Mashilo et al., 2018). Currently, twelve categories of cucurbitacins have been identified (Chen et al., 2005).

**Figure 1:**
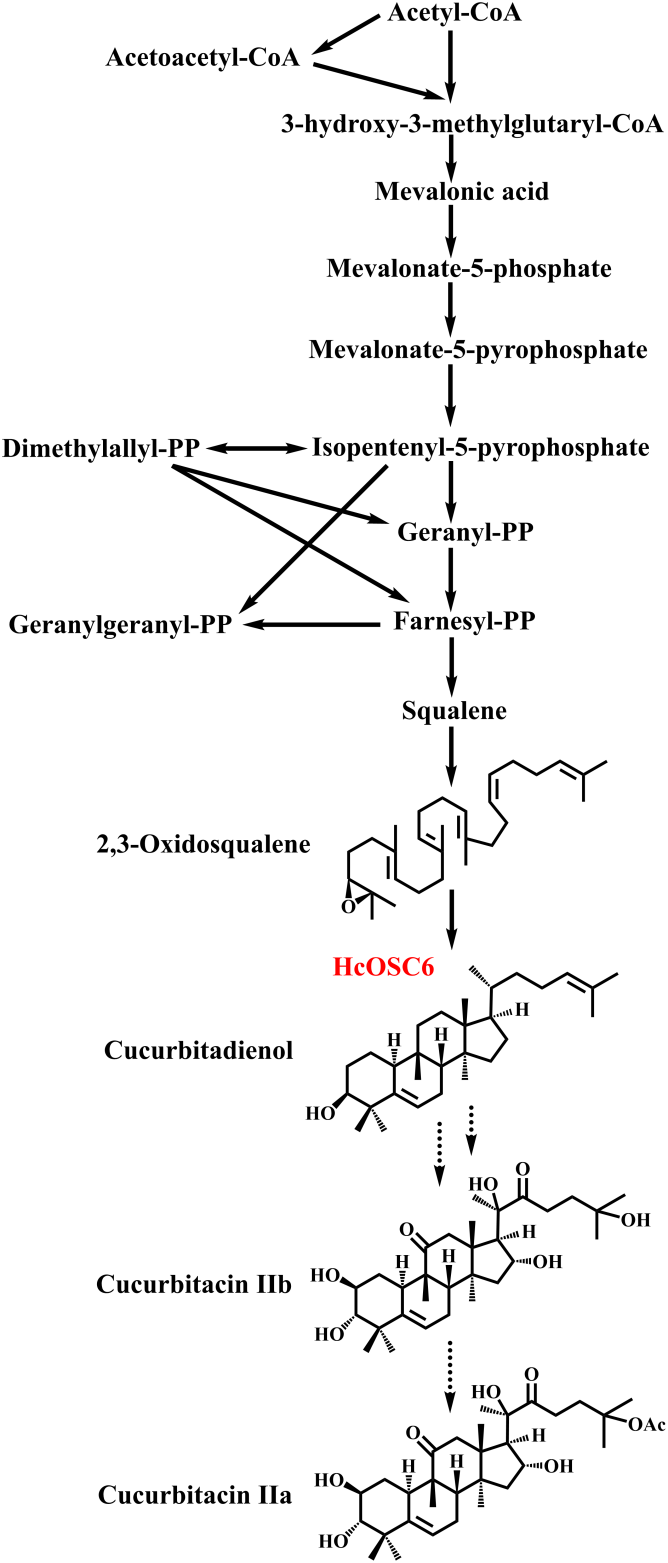
The biosynthetic pathway of cucurbitacins in *H. chinensis*.

In traditional Chinese medicine, cucurbitacin F has potential pharmacological properties, could be found as dihydrocucurbitacin F, hexanorcucurbitacin F, oxocucurbitacin F, or their glycosidic forms (Chen et al., 2005). Moreover, *Hemsleya* plants are used as traditional Chinese herbs, which contain cucurbitacin IIa (CuIIa) and cucurbitacin IIb (CuIIb) involved in treating digestive and respiratory inflammations (**Figure 1**). However, a few studies have investigated the biosynthesis of CuIIa and CuIIb (Chen et al., 2022). Previous research has established that 2,3-oxidosqualene is cyclized to produce cucurbitadienol to further generate cucurbitacins (Shibuya et al., 2004). A variety of cucurbitacins is produced *via* metabolizing cucurbitadienol in cucurbit crops (Zhou et al., 2016; Cárdenas et al., 2019). Cucurbitadienol synthase is the first enzyme responsible for cucurbitacin biosynthesis in cucurbits, which branches off from sterol synthesis (Shibuya et al., 2004). These pathways result in cucurbitacins with different molecular structures, some of which possess the properties of hydroxylation, ketone functions, double bonds and acetylation (Greige-Gerges et al., 2007; Zhou et al., 2016). Various cucurbit crops synthesize several cucurbitacins, such as cucurbitacin A, cucurbitacin B, cucurbitacin C, cucurbitacin D, cucurbitacin E, cucurbitacin F and cucurbitacin I (Gamlath et al., 1988; El-Fattah, 1994; Chen et al., 2005; Bejar et al., 2011; Ku et al., 2017; Salmi et al., 2018; Haq et al., 2019; Wu et al., 2019).

There are a number of genes in the oxidosqualene cyclases (OSCs) family which catalyze the conversion of 2,3-oxidosqualene and its analogues, such as (3S,22S)-2,3:22,23-dioxidosqualene (Shan et al., 2005; Thimmappa et al., 2014; Hoshino, 2017). The OSCs found in animals, plants, and fungi are ubiquitous (Shibuya et al., 2007; Mitsuguchi et al., 2009; Miettinen et al., 2018; Takase et al., 2019; Lertphadungkit et al., 2022). Approximately 100 triterpenoid skeletons are generated by OSCs in plants, contributing to natural terpene diversity (Xu et al., 2004; Suzuki et al., 2007; Wang et al., 2022). A total of 170 OSCs were functionally characterized from plants by May 2020, the majority of which came from non-model plant species (Ghosh, 2016; Sun et al., 2019). While OSCs differ in origin and function, their amino acids sequences are highly conserved. Compounds like triterpenoids possess high chemical complexity, making their synthesis challenging. As a result, the study of plant biosynthetic pathways proves to be of great importance and may yield opportunities for an increase in the production of triterpenoid compounds (Kumar et al., 2022). Furthermore, OSCs have been explored as a potential target for developing plant-active triterpenoids. Additionally, molecular docking and site-directed mutagenesis have been combined to provide valuable information on OSC specificity (Wu et al., 2019; Song et al., 2021).

A previous study showed that substitutions, insertions, and deletions of bases can significantly affect OSC activity (Chen et al., 2021). Furthermore, the highest contents of cucurbitacin IIa (CuIIa) and cucurbitacin IIb (CuIIb) in tubers were 1.43 mg/g and 0.19 mg/g dw, respectively, and HcOSC6 was identified as the key enzyme for cucurbitacin production (Chen et al., 2022). However, no reports have been found on how HcOSC6 affects the cucurbitacins accumulation. Furthermore, molecular docking analysis has shown to be effective in predicting protein-substrate binding sites in the active pocket of proteins (Hans et al., 2004; Osmani et al., 2008; Gholivand et al., 2013; Wang et al., 2014). Therefore, we used molecular docking analysis, along with mutations of the predicted key residues, to identify the key amino acid residues of HcOSC6. Moreover, an analysis of site-directed amino acid residue mutations was conducted to determine what amino acid residues are responsible for cyclization.

## Materials and methods

### Plant materials

*Hemsleya chinensis* were harvested in Kunming, Yunnan Province, China (E 102.95618, W 25.178849). RNA was extracted from the different tissues immediately after harvesting and storing them in liquid nitrogen.

### Total RNA isolation and cDNA synthesis

The frozen *H. chinensis* samples were ground into fine powder in liquid nitrogen with a mortar and pestle, and about 0.05 g was taken in a 2 mL Eppendorf tube, and the total RNA of different tissues was extracted according to a modified CTAB method [2×CTAB buffer: 2% CTAB, 100 mM Tris-HCl (pH 8.0), 25 mM EDTA, 2 M NaCl]. The RNA was purified using the RNA purification kit, and RNA quality was determined by electrophoresis on 1.0% agarose gels, and the concentration was measured by NanoDrop^TM^ Ultra-micro UV-Visible spectrophotometer. High purity total RNA was used to synthesize full-length cDNA according to the FastKing RT Kit instructions.

### Cloning of *HcOSC6* and expression vector construction

The sequence of *HcOSC6* gene were obtained with reference to the annotation information of *H. chinensis* transcriptome (PRJNA879990). Amplification of full length *HcOSC6* cDNA with gene specific primers was conducted by PCR. The reaction system was prepared according to the instructions of Phusion^®^ Hot Start Flex 2X Master Mix. After the PCR product was cut and recovered, the pYES2^®^-Blunt Zero vector was ligated and transformed into *E. coli* DH5α competent cells. The target gene containing the homologous arm of the vector was amplified by constructing expression vector primers, and the PCR product was cut and recovered; the yeast expression vector pYES2 was digested with *Bam*HI, and the digested product was recovered. The recombinant plasmid pYES2-*HcOSC6* was obtained by recombining the vector and the target gene fragment with pEASY^®^-Basic Seamless Cloning and Assembly Kit. The recombinant plasmid pYES2-*HcOSC6* was transformed into DH5α competent cells and cultured overnight on a LB solid plate containing 100 mg·L^-1^ Amp. The monoclonal colonies were selected for PCR test, and the positive results were used for sequencing.

### Molecular docking

AlphaFold2 provide the first computational method that can regularly predict protein structures with atomic accuracy even in cases in which no similar structure is known (Jumper et al., 2021). Protein structure models of HcOSC6 was generated using AlphaFold2. The purpose of this evaluation is to determine the stereochemical quality of the 3D model for HcOSC6, the model was estimated and evaluated (https://swissmodel.expasy.org/qmean/). The quality of the model was evaluated with Structural Assessment program (https://swissmodel.expasy.org/assess/). The Ramachandran plot visualizes the energetically favored regions of proteins backbone dihedral angles against amino acid residues. The protein model of HcOSC6 was docked with the 2,3-oxidosqualene by AutoDock4 (Morris et al., 2009). All molecular structures were visualized using PyMOL (Sidote et al., 2008).

### Site-directed mutagenesis in *E. coli*

Alanine was substituted for each of the predicted candidate amino acid residues of HcOSC6 to clarify the catalytic activity of these residues. The seventeen candidate amino acid residues were replaced with Ala for analysis. The PCR-based site-directed mutagenesis was performed using the primers shown in **Supplementary Table 1** using the expression plasmids for HcOSC6. PCR mixtures contained the following components: PrimeSTAR MAX Premix, 0.1 μM of each primer, 20 ng of the plasmid template, and double-distilled H_2_O to a final volume of 50 μL. The PCR conditions were as follows: 98° for 3min, 35 cycles of 98° for 10 s, 58° for 30s, 72° for 3min30s, and a final extension at 72° for 5min. All mutant gene were verified by DNA sequencing.

### Extracted product from GIL77 strain

To select the transformants, solid synthetic complete medium (SC-Ura) without uracil was used. Transformants were then cultured in SC-Ura medium supplemented with 2% glucose with 220 rpm shaking for 2 days at 30°C. A collection of cells was conducted and incubated with 2% galactose instead of glucose in 20 mL SC-Ura medium for 12 h at 220 rpm shaking at 30°C. The incubated cells were resuspended in 0.1M potassium phosphate buffer (pH 7.0) with 2% glucose at 30°C for 1 day at 220 rpm. Following collection of yeast cells, lysates of 20% KOH and 50% EtOH were prepared by ultrasonic extraction for 1 h or by refluxing for 5 min with 20% KOH and 50% EtOH. Hexane was used to extract the supernatant three times. The extracts were combined and evaporated by rotary evaporation. The product extracted in yeast GIL77 brewer was dissolved in 200 μL of methanol solution (purity 99.9%). The obtained sample was filtered through a 0.22 μm organic phase filter and detected with an Agilent 1260 HPLC. Cucurbitadiol yield (μg/g) is defined by the following equation: [(G/S) × V]/OD value. Where G is the volume of the incoming detector sample, S is the volume of the total sample. Cucurbitadiol conversion efficiency (%) = Cucurbitadiol yield/GIL77 yeast culture time.

### Phylogenetic analysis

A Muscle algorithm was used to align the OSCs protein sequences. MEGA7 software was used to construct a neighbor-joining tree based on 1000 bootstrap replications using default parameters.

## Results

### 3D Model construction of HcOSC6

Cucurbitacin F is commonly derived from cucurbitadienol. Previous studies have explored that *HcOSC6* was clustered with characterized cucurbitadienol synthases from cucurbitaceae, and its expression pattern was regarded as responsible for the accumulation of cucurbitadienol, indicating *HcOSC6* was a cucurbitadienol synthase involved in cucurbitadienol biosynthesis (Chen et al., 2022).

**Figure 2:**
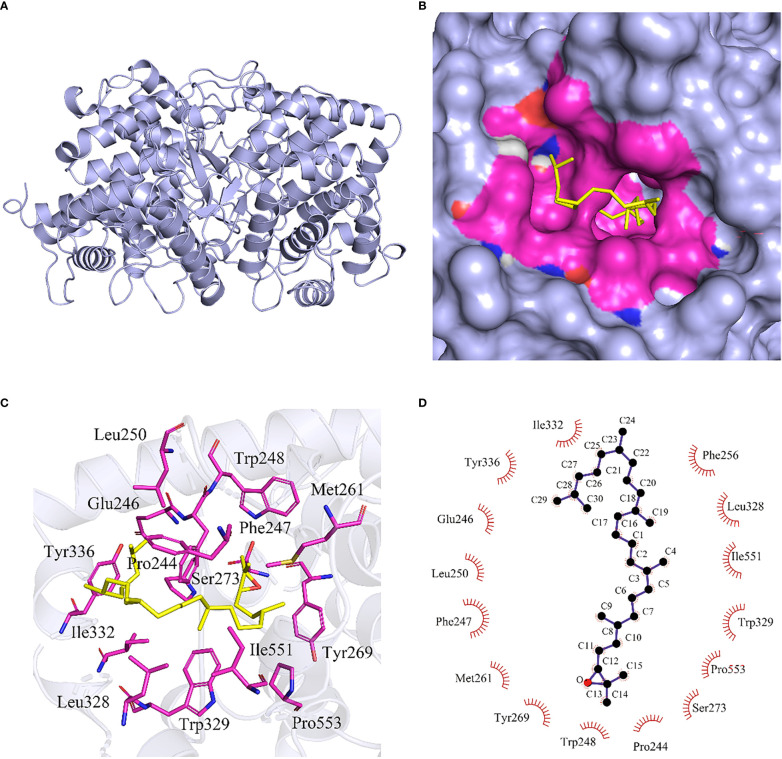
Molecular docking of 2,3-oxidosqualene to DNA binding site of HcOSC6. **(A)** The HcOSC6 nucleotide sequence was translated into protein sequence, highly accurate protein structure prediction with AlphaFold2, save its PDB file and visualize it using PyMOL software, and the predicted three-dimensional (3D) protein colored light purple is shown in cartoon representation. **(B)** Surface model representing the interacting site HcOSC6 with 2,3-oxidosqualene. The protein-colored light purple is shown in surface representation. The 2,3-oxidosqualene binding site containing interacting residues colored differently is in surface representation. The bound 2,3-oxidosqualene in stick representation colored blue with oxygen and hydrogen atoms colored red and white, respectively. Yellow color represented ligand and rosy color represented interacting amino acids. **(C)** Protein-ligand interaction plot of 2,3-oxidosqualene bound to HcOSC6. The magenta stick indicated the amino acid residue that interacts with the 2,3-oxidosqualene at a distance of 4Å, and hydrophobic bonds were formed between the amino acid residues and the ligand. **(D) **Two-dimensional surface representation of the binding of 2,3-oxidosqualene and key residues with hydrophobic interaction. The 2,3-oxidosqualene was shown in ball-and-stick representation. The black balls showed carbon atoms whereas the red balls showed the oxygen atoms. The residues involved in non-bonding interactions were shown as red bristles. The 2,3-oxidosqualene had hydrophobic interactions with I332, Y336, E246, L250, F247, M261, Y269, W248, P244, S273, P553, W329, I551, L328 and F256.

To identify the key amino acid substitutions in HcOSC6 that affect the cyclization reaction and contribute to differences in enzyme activity, 3D model of HcOSC6 was constructed using AlphaFold2. Therefore, the first step in our study was to carry out computer modeling and molecular docking analysis to determine the biochemical role of HcOSC6. The full-length protein sequence of the HcOSC6 gene is from *H. chinensis*, which belongs to the Cucurbitaceae family (Chen et al., 2022). Based on the protein sequence of *H. chinensis*, the first 3D structure of HcOSC6 in *H. chinensis* was predicted by training the model (**Figure 2A** and **Supplementary Figure 1A**). The 3D structure of HcOSC6 was constructed and saved as a PDB file for visualization using PyMOL. Ramachandran plot results in **Figure S1B** showed that 96.74% of amino acids of HcOSC6 were located in region where backbone dihedral angles were energetically favored. Furthermore, the QMEANDisCo global score was 0.76 ± 0.05, and that all QMEAN Z-scores were higher than −0.41, indicating that this predicted model of HcOSC6 was high quality (**Supplementary Figures 1B, D**). The charge distribution of HcOSC6 was illustrated in **Figure S1C**, with an electrostatic potential difference ranging from −60.80 to +60.80. The TMH domains of HcOSC6 displayed a neutral potential (white), while extrinsic and periplasmic regions displayed the negative (red) and positive (blue) potentials (**Supplementary Figure 1C**).

Model quality is measured by the agreement between the score of the model and the values of the resultant structures from a high-resolution experiment. Comparing the QMEAN Z-score of the target protein with those of the non-redundant protein structures, the results showed that the score was close to 0 (**Supplementary Figure 2A**). For models located in dark zones, scores were less than 1, while those outside of the dark zones could be either 1 < the Z-score < 2 or Z-score > 2. Good models are often located in the dark zone (**Supplementary Figure 2A**). The HcOSC6 model structure was analyzed, and the model generated by Phyre2 showed that the HcOSC6 membrane protein alpha helix structure was concentrated at the N-terminal end (**Supplementary Figure 2B**).

### Molecular docking and active site analysis of HcOSC6

**Figure 3:**
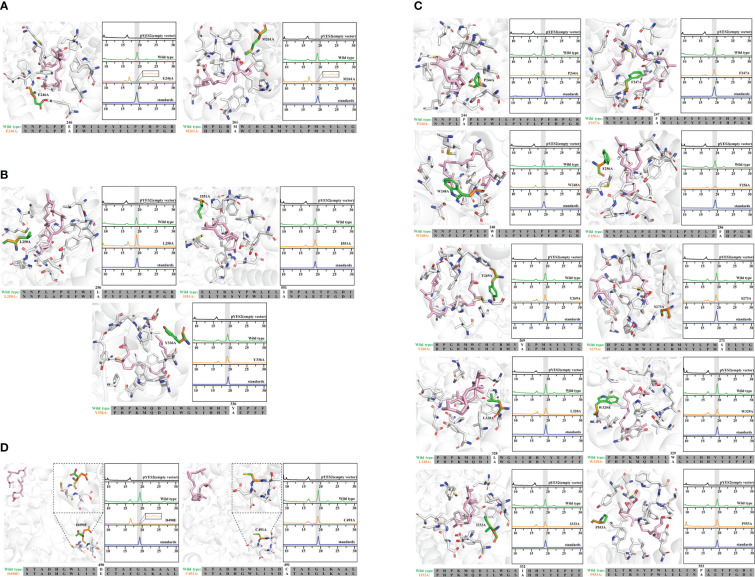
Molecular docking and site-directed mutagenesis of 2,3-oxidosqualene cyclase (HcOSC6). **(A)** Model docked view of HcOSC6 and 2,3-oxidosqualene (ligand); HPLC analysis of yeast extracts expressing the wild and mutant HcOSC6 (E246A, M261A). **(B)** Model docked view of HcOSC6 and 2,3-oxidosqualene (ligand); HPLC analysis of yeast extracts expressing the wild and mutant HcOSC6 (L250A, I551A and Y336A). **(C)** Model docked view of HcOSC6 and 2,3-oxidosqualene (ligand); HPLC analysis of yeast extracts expressing the wild and mutant HcOSC6 (P244A, F247A, W248A, F256A, Y269A, S273A, L328A, W329A, I332A and P553A). **(D)** Model docked view of HcOSC6 and 2,3-oxidosqualene (ligand); HPLC analysis of yeast extracts expressing the wild and mutant HcOSC6 (D490E and C491A).

It has been demonstrated that site-directed mutagenesis and substrate structural modification are two approaches that could be utilized to identify the key residues and active sites in plant OSCs. Firstly, HcOSC6 was cloned into the pYES2 vector, containing a truncated HMGR gene, and then transformed into yeast to determine the biochemical roles of HcOSC6. Additionally, HcOSC6 is an oxidosqualene cyclase that has produced the greatest yield of cucurbitadienol (Chen et al., 2022). In order to better understand how amino acids substitutions in HcOSC6 affect the processes of 2,3-oxidosqualene cyclization and lead to different products, the binding mode of HcOSC6 with 2,3-oxidosqualene was analyzed, and a docking experiment with the substrate 2,3-oxidosqualene was conducted (**Figure 2B**). In this study, the binding of 2,3-oxidosqualene to HcOSC6 was performed *via* Autodock4, which showed residues near the active site pocket that may be important for its activity (**Figure 2C**). A total of 15 candidate amino acid residues (E246, M261, L250, Y336, I551, P244, F247, W248, F256, Y269, S273, L328 W329, I332 and P553) possibly involved in the cyclization reaction between 2,3-oxidosqualene and HcOSC6 were identified (**Figure 2D** and **Supplementary Figure 3**). The identification of critical residues to protein structure, function, and stability can be quickly accomplished using combinatorial libraries of alanine-substituted proteins. Using alanine substitutions, each amino acid side chain is examined for its contribution to the protein function (Morrison and Weiss, 2001). We aligned the structures of HcOSC6 to identify residues important for substrate recognition. These distinct amino acids bind to their substrate in pockets were highlighted in **Figure 3**.

In addition, knowledge of conserved regions is limited in comparison to the highly conserved structure of plant OSC. While DCTAE has not been fully validated for its function, and the key residues participating in the polycyclization cascade in the early stage (rings A/B/C) are rare, however, multiple sequence alignments found that OSCs contain a highly conserved “DCTAE” motif, which is responsible for binding substrates and initiating polycyclization of 2,3-oxidosqualene (Abe and Prestwich, 1995; Thoma et al., 2004). Furthermore, previous research has established that the DCTAE motif of OSC is relatively conserved, while HcOSC6 features showed that it is worth mentioning that these residues participated in cyclization reaction (Chen et al., 2022). Therefore, these above residues were selected for further site-directed mutagenesis experiment to determine whether they were key amino acids for cucurbitadienol production in HcOSC6.

### Changes in key amino acid residues alter conversion efficiency of 2,3-oxidosqualene to cucurbitadienol in yeast

**Figure 4:**
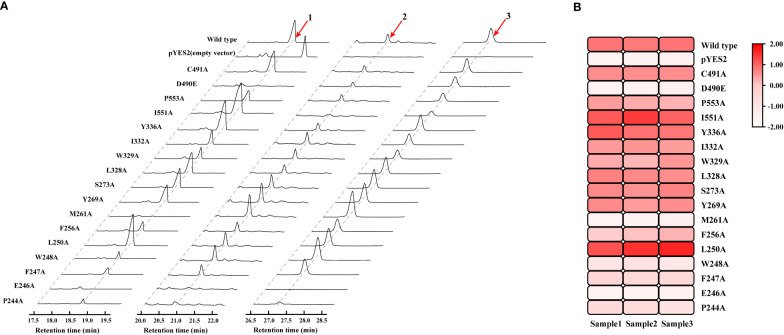
Mutagenesis-based alterations show residues that control substrate affinity of HcOSC6 and the catalysis activity for HcOSC6. **(A)** GC-MS analysis of yeast extracts expressing the wild type and mutant HcOSC6. Peak 1 is cucurbitadienol; Peak 2 is 25-Hydroxycholesterol,3-trimethylsilyl; Peak 3 is 5β-Cholestane-3α,7α,12α,24α,25-pentol. **(B)** Heatmap of the cucurbitadienol conversion rates related to HcOSC6. The degree of redness indicates the conversion rate.

HcOSC6 were cloned from *H. chinensis* and a mutant plasmid was constructed in *E. coli*. The primers involved in the construction of the mutant were shown in **Supplementary Table 1**. The mutant was expressed in the LSS-deficient yeast strain GIL77, which produced cucurbitadienol. The cyclization products were determined, which used gas chromatography-mass spectrometry (GC-MS) to analyze yeast cell extracts. Furthermore, a galactose-responsive promoter GAL1, which was repressed by glucose, controlling the expression of OSC. Additionally, lanosterol was the precursor for ergosterol biosynthesis, which was the main sterol in yeast. A variety of yeast strains containing OSC constructs grew when glucose and ergosterol were added exogenously. The *Saccharomyces cerevisiae* strain with the target gene could extract three terpenoids, and the GC-MS identification analysis TIC plot showed three peaks at around 18.701, 20.801 and 27.118 min, respectively, of which the peak at 18.701 min was cucurbitadienol, followed by two compounds, peak 2 and peak 3, which were 25-Hydroxycholesterol,3-trimethylsilyl and 5β-Cholestane-3α,7α,12α,24α,25-pentol (**Figure 4A** and **Supplementary Figure 4**). The GC-MS identification analysis EIC diagram is shown in the **Supplementary Figure 5**.

**Figure 5:**
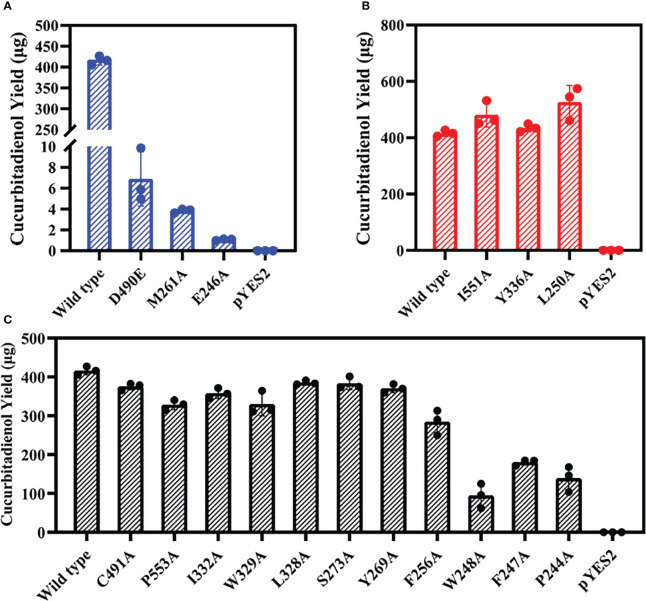
Total activities for the mutants obtained were evaluated against that of the wild-type HcOSC6 (100%). Data represent mean ± SD of three biological replicates. **(A)** Cucurbitadienol yield for the mutants D490E, M261A and E246A. **(B)** Cucurbitadienol yield for the mutants I551A, Y336A and L250A. **(C)** Cucurbitadienol yield for the mutants C491A, P553A, I332A, W329A, L328A, S273A, Y269A, F256A, W248A, F247A and P244A.

Mutation yields analysis was determined by monitoring the cucurbitadienol contents using 2,3-oxidosqualene as the substrate. The thin layer chromatography showed that no bands was observed for the cucurbitaenol compounds E246A and M261A while the replacement of E246 and M261 of HcOSC6 with Ala (**Supplementary Figure 6**), and were detected at very low levels of cucurbitaenol, which were 0.27% and 0.95% using HPLC (**Figures 3A**, **4B**, **5**). However, substitution of three candidate amino acid residues (L250A, Y336A and I551A) increased the production of cucurbitadienol at 105% to 127% compared with those in wild type (**Figures 3B**, **4B**, **5**). Other mutation sites (P244A, F247A, W248A, F256A, Y269A, S273A, L328A, W329A, I332A and P553A) led to decrease the contents of cucurbitadienol around 22.64% to 92.59% (**Figures 3C**, **4B**, **5**). The heat map of the above mutant affecting the cucurbitadienol conversion rate of HcOSC6 was shown in **Figure 4B**. The cucurbitadienol conversion efficiency of mutants E246A and M261A can be significantly lower than that of wild type.

To assess whether the amino acid substitution of conserved DCTAE sequence also affects the activity of HcOSC6, we generated mutants HcOSC6-D490E and HcOSC6-C491A. Heterologous expression of GIL77 *Saccharomyces cerevisiae* strain showed that the site mutations of D490 to Glu led to extremely reduced to 1.67% of cucurbitadienol production (**Figures 3D**, **5A**), while the substitutions of C491A decreased cucurbitadienol accumulation (**Figures 3D**, **5C**). Additionally, previous studies showed that SgCbQ contains the DCTAE motif, which may be responsible for initiating the cyclization process (Abe and Prestwich, 1995), and for the cucurbitadienol synthase SgCbQ, mutants D486N/A and C487A/M generated compounds with CCC conformations, suggesting they were involved in substrate folding (Qiao et al., 2018; Chen et al., 2021), whereas those of E246 and M261 in HcOSC6 were newly identified in this study. Therefore, the residues E246, M261 and D490 were proven to stabilize the intermediates and affect the catalytic activity during cyclization reaction. It is known that the epoxide group of prefolded 2,3-oxidosqualene is protonated by the Asp in plant OSCs (Thoma et al., 2004; Ito et al., 2013). It is essential for the OSC activity to highlight the DCTAE region in mutagenesis studies. Additionally, EtAS mutants with D485N/E lost their activities, while C486A and C489A reduced the yield of dammarenediol-II (Ito et al., 2013).

### Phylogenetic tree construction

**Figure 6:**
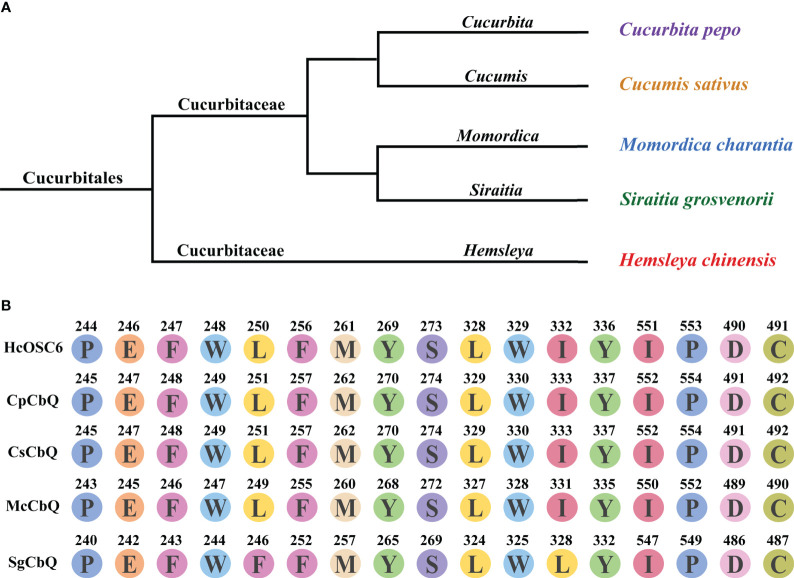
Divergence of HcOSC6 contributes to *Hemsleya* in Cucurbitales. **(A)** Phylogenetic relationship of five analyzed plant species, originated from the Cucurbitaceae family. **(B)** Summary of key residues in the selected sequences. Data was extracted from the OSCs alignment from five species. Functionally characterized enzyme names are labeled in black; Branch colors represent.

Cucurbitales have detailed understanding of OSC diversity, but other plant orders have little information. OSCs also have an unclear molecular origin and functional diversity during plant evolution. *H. chinensis* is a flowering plant from the *Hemsleya*, a genus in the Cucurbitaceae family. The evolutionary tree analysis was shown in **Figure 6**. The cavity pocket site of 2,3-oxidosqualene docked with HcOSC6 in *H. chinensis* was compared with the amino acid sequences of four species that can produce cucurbitadienol, and it was found that all plants except *S. grosvenorii* had highly conserved amino acid residue sites (**Figure 6B**). Then the phylogenetic analysis of the other five OSCs in the family of OSCs in *H. chinensis* showed that HcOSC1 clustered with the cycloartenol synthase subgroup, HcOSC2 and HcOSC3 clustered with the β-amyrin synthase subgroup, HcOSC4 clustered with the multifunctional triterpene synthase subgroup, and HcOSC5 clustered with the isomultiflorenol synthase subgroup. (**Supplementary Figure 7**). The nucleotide sequences of the six OSCs were analyzed for conserved motifs (**Supplementary Figure 8**), amino acid number, molecular weight and theoretical pI properties (**Supplementary Table 2**), hydrophilic hydrophobicity (**Supplementary Figure 9A**), and protein transmembrane structure (**Supplementary Figure 9B**).

## Discussion

Natural products derived from plants contain abundant active compounds, and have been utilized as herb medicines since ancient times (Grzech et al., 2023). Its abundant primary and secondary metabolites have made Chinese herbal medicine increasingly popular in recent years. A number of bioactive terpenoids are present in plants, with a wide range of bioactive properties and ecological potential (Amirzakariya and Shakeri, 2022; Darshani et al., 2022). Isopentenyl diphosphate (IPP) and dimethylallyl diphosphate (DMAPP) are key precursors for triterpene biosynthesis, generated *via* mevalonate pathway (MVA) and methylerythritol phosphate pathway (MEP), respectively. Then, squalene is generated by sequentially catalyzing IPP and DMAPP. The oxidation of squalene forms 2,3-oxidosqualene by squalene epoxidase. The 2,3-oxidosqualene is catalyzed into multicyclic structures, which is the branching point between metabolites of steroids and triterpenoids (Nagegowda and Gupta, 2020). Furthermore, cucurbitacins found in Cucurbitaceae plants possess a variety of pharmacological functions, including anti-cancerous and anti-inflammatory effects (Chen et al., 2012). In addition, the biosynthesis pathways of curcurbitacins are gradually being revealed in *S. grosvenorii*, which belongs to the Cucurbitaceae family (Dai et al., 2015; Lertphadungkit et al., 2021). To gain a better understanding of the biosynthetic pathways of a majority of the pharmacologically active compounds in *H. chinensis*, we characterized several genes involved in this pathway in our previous study. Moreover, we functionally characterized key SEs, OSCs, and ATs for the first time in *H. chinensis*, implying that they are involved in the accumulation of cucurbitacins (Chen et al., 2022).

OSCs are the rate-limiting enzymes involved in triterpene biosynthesis and substrates binding, initiates the reaction with protonation, cyclization and rearrangement, and terminates the reaction by deprotonation or water capture (Corey and Virgil, 1991; Liang et al., 2022). However, the detailed mechanisms of this process are not fully understood. Molecular skeletons could be classified as sterols or triterpenes based on conformational analysis. Genes encoding enzymes for pentacyclic triterpene skeletons *via* C-C-C conformation dammarenyl cation paths were found in one category, while genes encoding enzymes for tetracyclic skeletons were found in the other (Thimmappa et al., 2014). Triterpene diversification begins with the cyclization of 2,3-oxidosqualene by OSC in plants (Al-Harrasi et al., 2021), thus resulting in more than 100 kinds of triterpene skeletons (Shang and Huang, 2019). In this study, metabolomic studies showed that six OSCs in *H. chinensis* compete for the same substrate to produce various triterpenes. A total of six OSCs were annotated in this transcriptome, named HcOSC1-HcOSC6. As previously reported, HcOSC6 is the main enzyme involved in the synthesis of cucurbitacins in *H. chinensis*, catalyzing 2,3-oxidosqualene into cucurbitadienol, which serves as the starting substrate for CuIIa and other cucurbitacins (Chen et al., 2022). Additionally, previous study found that reported 4 novel OSCs from *M. charanti*, related to McCBS, McIMS, McBAS and McCAS (Takase et al., 2019). Moreover, three novel OSCs from *T. cucumerina* L. were reported in recent studies, TcBAS, TcLAS, and TcCAS, which display different activities (Lertphadungkit et al., 2022). Thus, diverse OSCs contribute to the diversity of triterpenoids in *Hemsleya* plants.

An effective approach to identifying the catalytic mechanisms of various enzymes in plants has been to use protein homology modeling and site-directed mutagenesis. Previous reports have proposed several strategies to enhance the number of active compounds, including functionally modifying OSCs by altering the amino acid residues associated with triterpene product specificity (Xue et al., 2018). Therefore, to better understand the mechanisms of cucurbitacins formation and increasing the amount of active cucurbitacins produces great benefits. In this study, we identified the key active site residues controlling cucurbitadienol accumulation specificity; these amino acid residues were critical for both product and substrate affinity. Moreover, a single mutation of HcOSC6-L250A, HcOSC6-Y336A and HcOSC6-I551A resulted in a highly conversion from 2,3-oxidosqualene to cucurbitadienol, indicating three single-residue alterations greatly improved substrate affinity of HcOSC6 (**Figure 4**). Phylogenetic tree analysis showed that HcOSCs were predominantly divided into different branches (**Supplementary Figure 7**), explaining their significant similarity between the structure of cucurbitacins and steroidogenic triterpenes. Some amino acids located at the active center of OSCs are responsible for the catalytic activity. Therefore, it is crucial to understand how amino acids are positioned, how OSCs function, and how they catalyze. Our results showed that the key amino acids in HcOSC6 were identified with the help of the newly developed AlphaFold2 software. Site-directed mutagenesis experiments revealed that two single mutants, HcOSC6-E246A and HcOSC6-M261A, significantly lowered cucurbitadienol production. Furthermore, the DCTAE sequence is a highly conserved sequence that can be used to preliminarily identify OSCs, while it plays a crucial role in substrate binding and the initial cyclization of substrates. It has been reported that the DCTAE motif is highly conserved in eukaryotic OSCs, initiating the polycyclization of squalene epoxide (Ito et al., 2013). The acidic carboxyl residue Asp in this motif releases protons to attack on the terminal epoxide ring of 1, which triggers a cascade of the ring-forming reaction; Furthermore, the acidity of Asp455 in the DCTAE sequence is increased by forming hydrogen bonds with Cys456 and Cys533 (Thoma et al., 2004). Ito et al. (2013) constructed four mutants of EtAS (D485N, D485E, C486A and C564A), mutants D485N and D485E were inactive. After multiple sequence comparison, our results showed that D490 in the highly conserved DCTAE sequence in HcOSC6 was replaced with E. It was hypothesized that the amino acid change would increase the carbon chain length of aspartate, distancing D490 from C491, making the C491 cyclization mechanism weaker and hydrogen bonds less likely to form, thus changing the acidity of D490 and leading to the inactivity of HcOSC6. In this study, the D490 site was substituted with Glu to determine its function, indicating HcOSC6-D490E mutants showed a significant decrease in cucurbitadienol accumulation. These findings suggested that this position might be responsible for the conformational changes caused by cucurbitadienol formation (**Figure 3D**). Thus, studying key amino acids is important to understand the function of HcOSC6. This is done by combining model construction with molecular docking and site-directed mutagenesis. We constructed a HcOSC6 model to verify which residues are responsible for the functional specificity of HcOSC6. Taken together, these findings provided new insights into the biosynthesis for the diversity of cucurbitacins in *H. chinensis*.

## Conclusion

In conclusion, a reliable 3D model of HcOSC6 was first developed to explore the potential function of the active site using AlphaFold2. Molecular docking and site-directed mutagenesis were used to investigate the catalytic mechanism of HcOSC6. Several potential amino acid residues were critical for HcOSC6, referred to the E246, M261 and D490, that could be responsible for the cyclization of cucurbitadienol. Furthermore, the amino acid sequences of four plants that can produce cucurbitadienol, were compared with the cavity pocket site of 2,3-oxidosqualene docked with HcOSC6 in *H. chinensis*, indicating all plants had highly conserved sequence except *S. grosvenorii* and diverse OSCs result in different triterpenoids. Our findings not only comprehensively characterize three key residues that are potentially useful for producing cucurbitacins, but also provide insights into the significant role they could play in metabolic engineering.

## Data availability statement

The data presented in the study are deposited in the National Center for Biotechnology Information (NCBI) repository, accession number PRJNA879990.

## Author contributions

YZ and C-XZ conceived and designed the experiments. XL, GC, Q-QG, C-FX, C-XY and Y-YS performed the experiments. X-NL, G-HZ, Y-LL and S-CY analyzed the data. YZ, XL and C-XZ wrote the article. All authors contributed to the article and approved the submitted version.
