# Supplementary material for: Site-directed mutagenesis identified the key active site residues of 2,3-oxidosqualene cyclase HcOSC6 responsible for cucurbitacins biosynthesis in Hemsleya chinensis
Source: Front Plant Sci. 2023 Mar 28;14:1138893. doi: 10.3389/fpls.2023.1138893 (PMC10086137; doi:10.3389/fpls.2023.1138893)
Supplement: Supplementary file 1 [file DataSheet_1.docx]

**
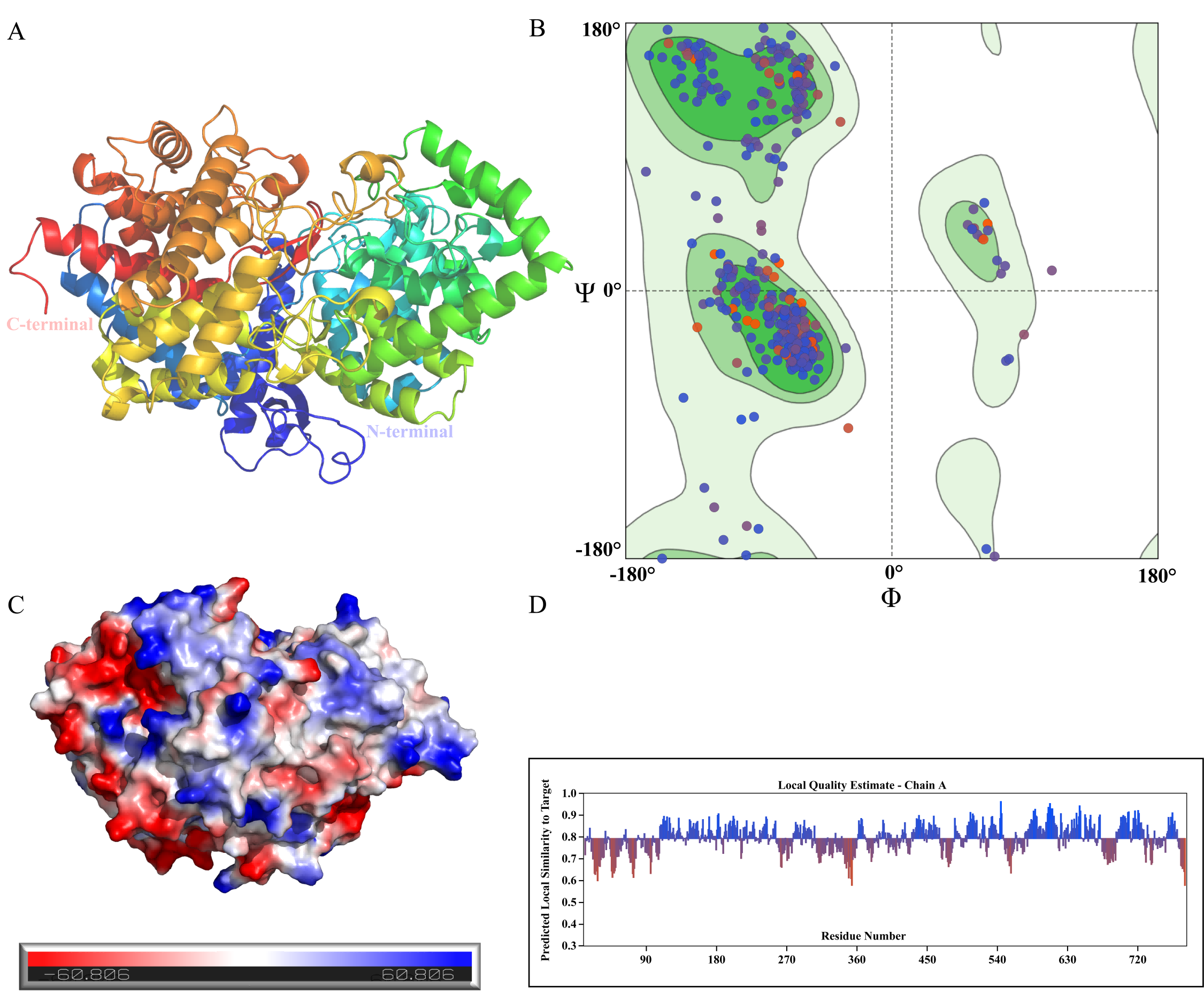
**

**Supplementary Figure 1.** 3D model construction and evaluation.

(A) 3D model of HcOSC6 constructed by the AlphaFold2.

(B) The Ramachandran plot paragraph of HcOSC6.

(C) Overall charge distribution of HcOSC6 with the electrostatic potential difference from -60.8 to +60.8.

(D) The QMEAN Z-cores for HcOSC6 3D model evaluation.

**
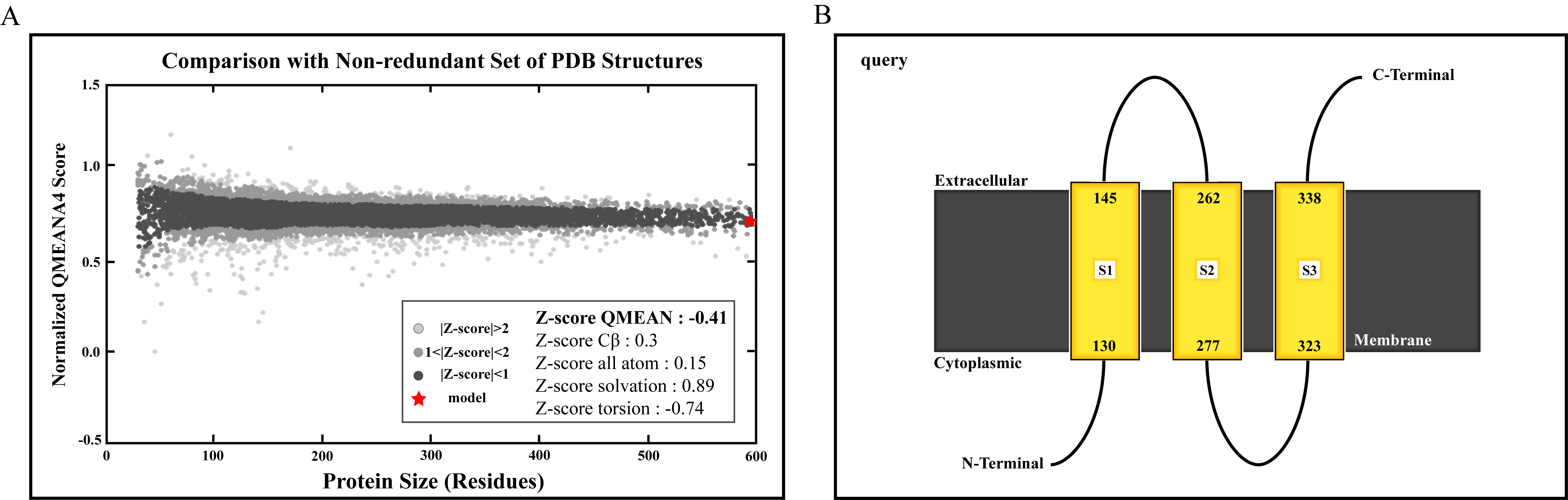
**

**Supplementary Figure 2.** Structural prediction and analysis of HcOSC6.

(A) Normalized QMEAN score of theoretical 3D structure for HcOSC6 model created with SWISS-MODEL server.

(B) Transmembrane region of HcOSC6 predicted by Phyre2.

**
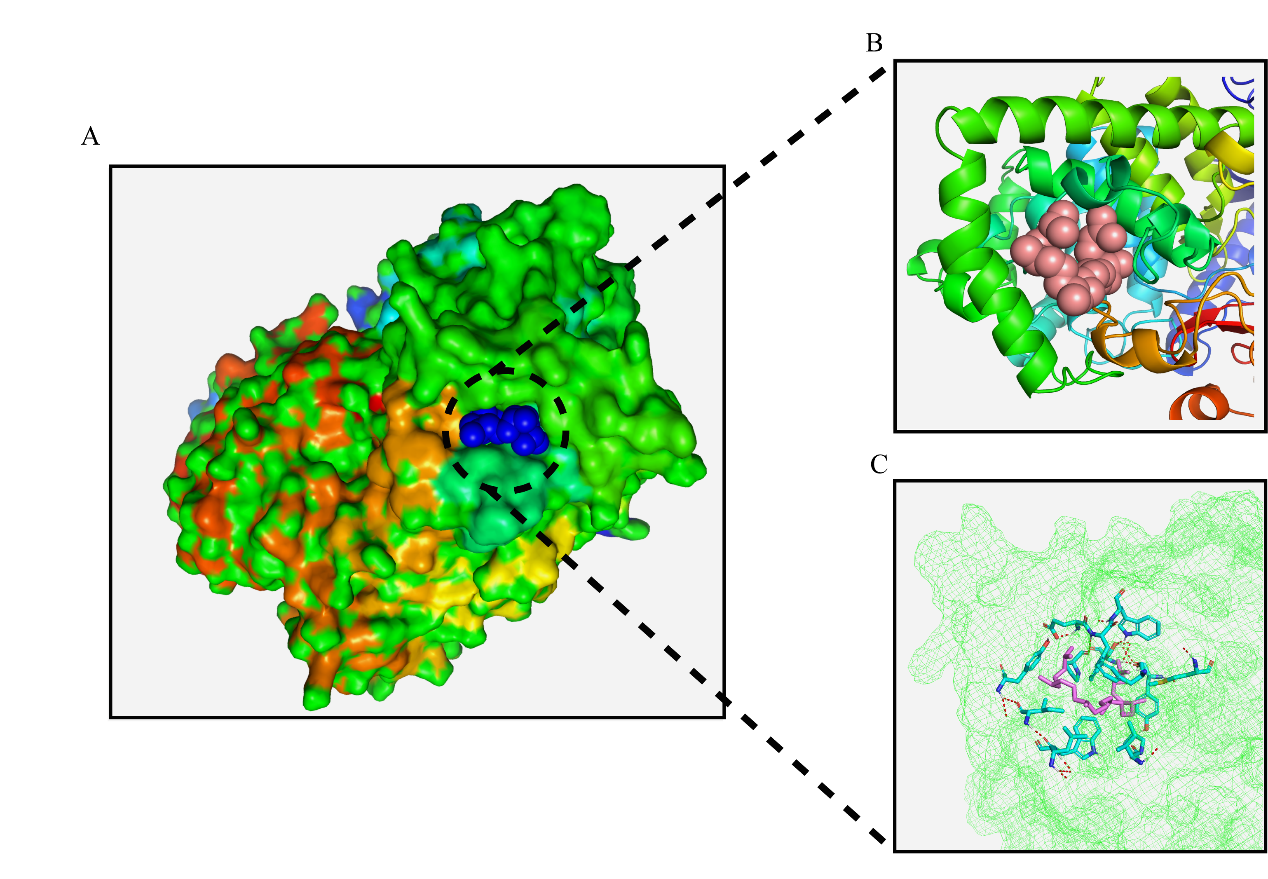
**

**Supplementary Figure 3.** Schematic representation of substrate 2,3-oxidosqualene interaction with active site residues of HcOSC6 calculated using the PyMOL program.

(A) The 3D structure of HcOSC6 binding 2,3-oxidosqualene shown as surface.

(B) The cartoon representation of the HcOSC6-2,3-oxidosqualene.

(C) Dots and mesh representation of the binding of HcOSC6 and key residues (I332, Y336, E246, L250, F247, M261, Y269, W248, P244, S273, P553, W329, I551, L328 and F256) with hydrophobic interaction.

**
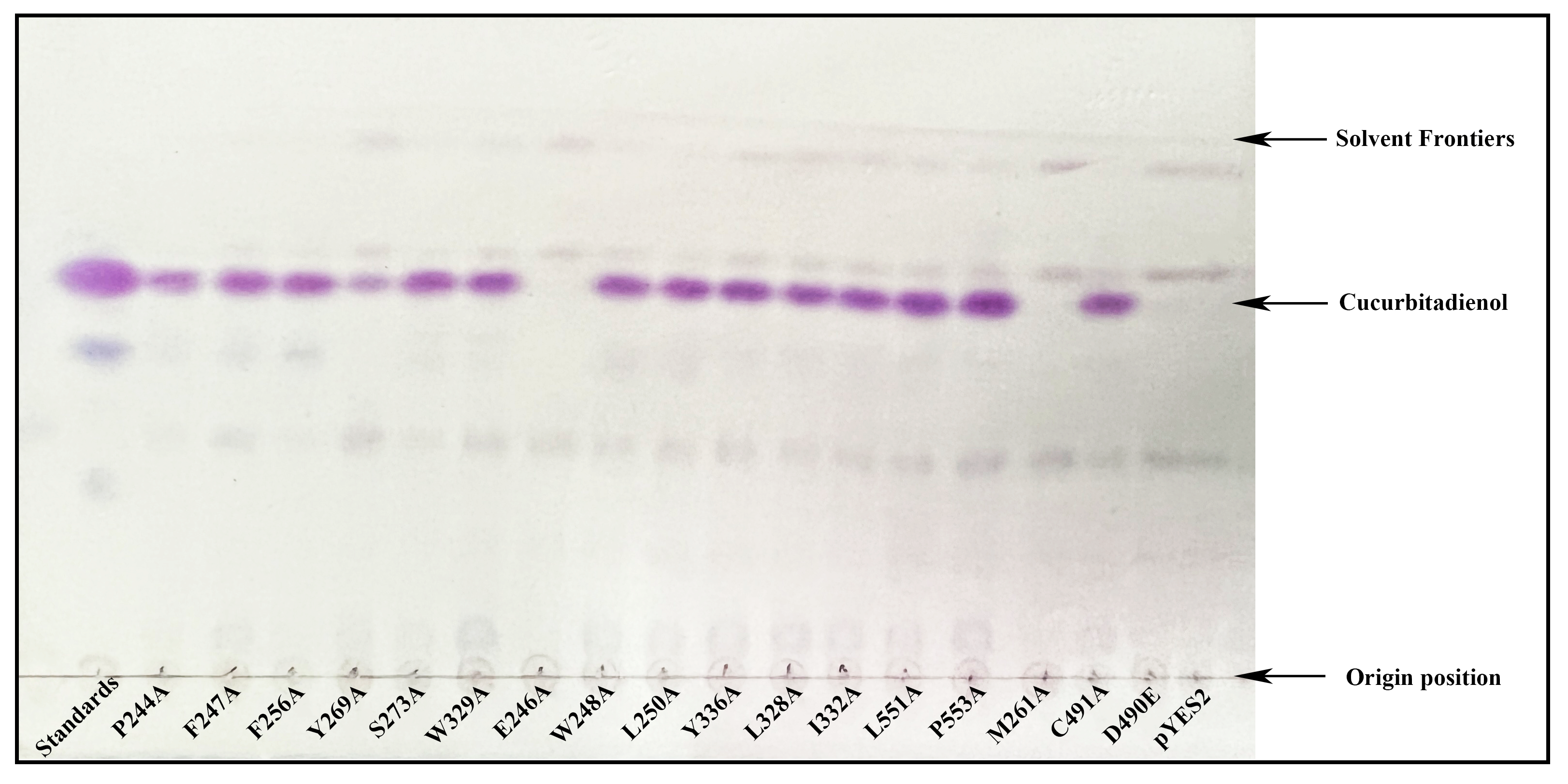
**

**Supplementary Figure 4.** Thin layer chromatography (TLC) of ethyl acetate extract of GIL77 yeast isolate.

The yeast cells of GIL77 yeast were decomposed with the same volume of 20% KOH, 98°C, for 5 min, and then extracted with ethyl acetate in the same volume three times to obtain the extract, which was unfolded with a spreading agent using petroleum ether and ethyl acetate (6:1) and developed on silica gel TLC plates using ethanol, sulfuric acid and a mixture of ethanol: sulfuric acid (90:10) was heated with a heater on the silica gel TLC plate soaked with the stain, and species-specific molecules present in the extract of the GIL77 yeast strain were observed.


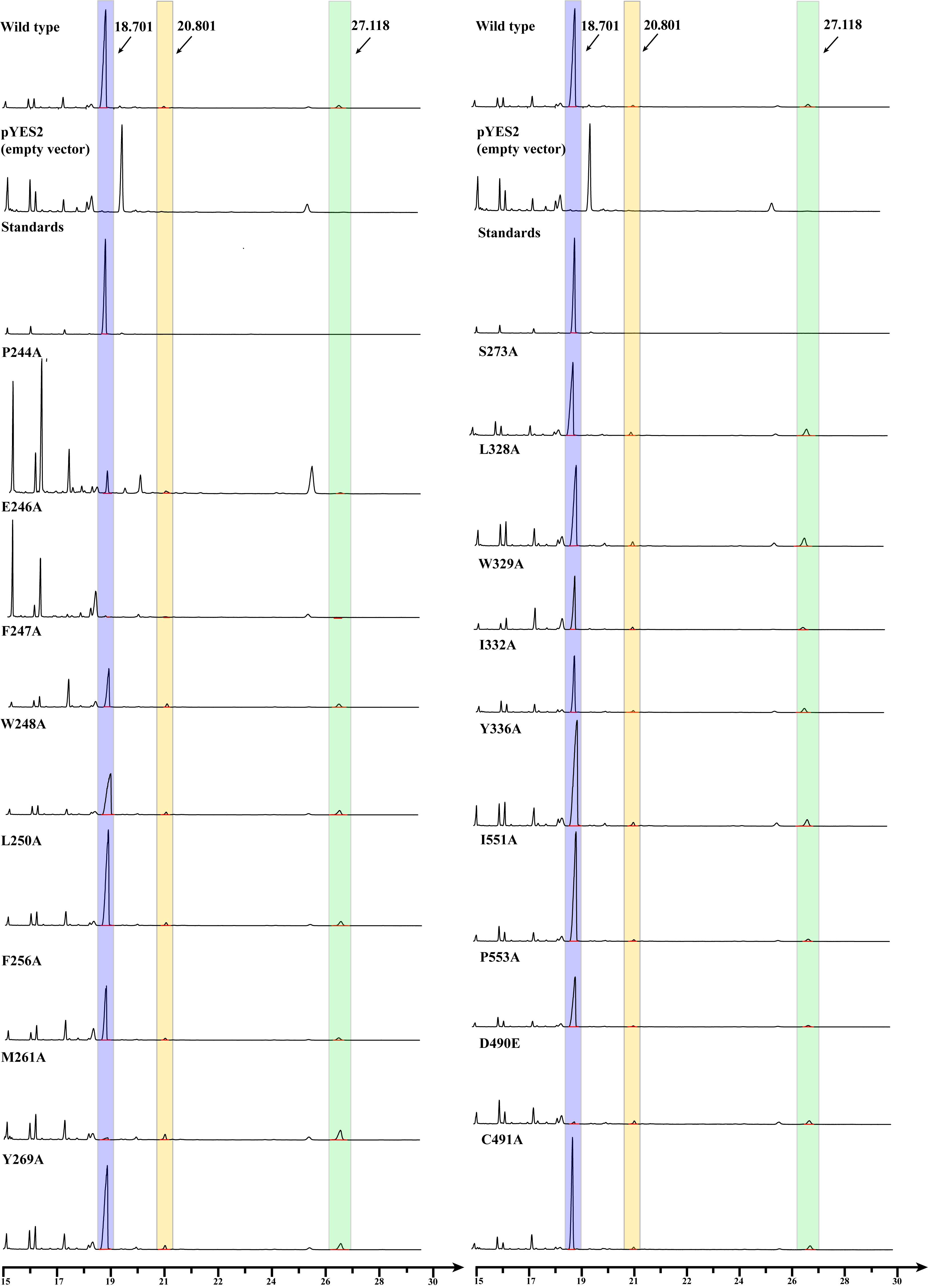


**Supplementary Figure 5.** GC-MS total ion chromatogram (TIC) of extracts from yeasts harbouring mutant pYES2-HcOSC6 (I332A, Y336A, E246A, L250A, F247A, M261A, Y269A, W248A, P244A, S273A, P553A, W329A, I551A, L328A, and F256A) and pYES2 (empty vector) compared with cucurbitadienol standard.

The purple label shows cucurbitadienol with a peak time of 18.701 min; The yellow label shows 25-Hydroxycholesterol,3-trimethylsilyl with a peak time of 20.801 min; The green label shows 5β-Cholestane-3α,7α,12α,24α,25-pentol with a peak time of 27.118 min.


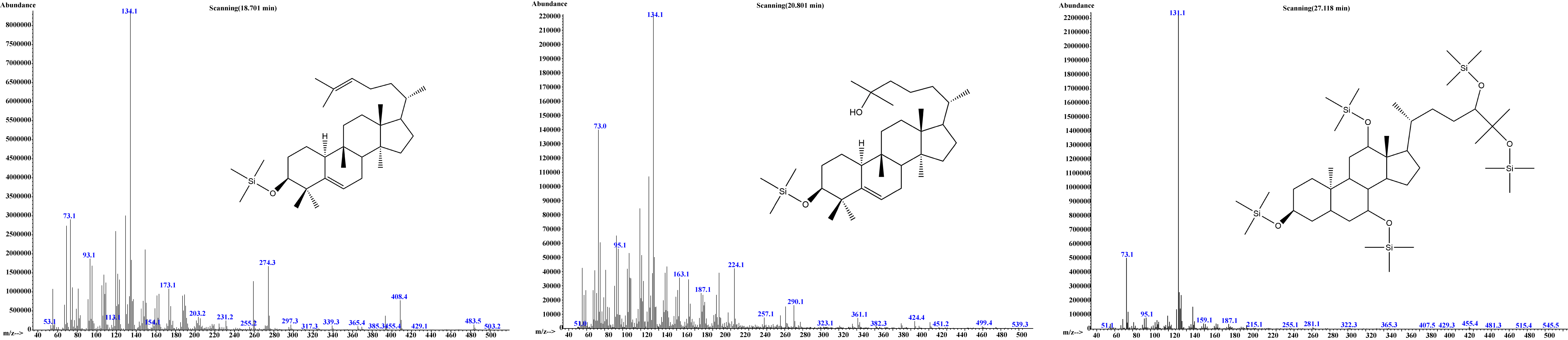


**Supplementary Figure 6.** Extracted ion chromatogram (EIC) of three corresponding compounds in Supplementary Fig.5 by GC-MS.

The compound with a peak time of 18.701 min (left); The compound with a peak time of 20.801 min (middle); The compound with a peak time of 27.118 min (right).

**
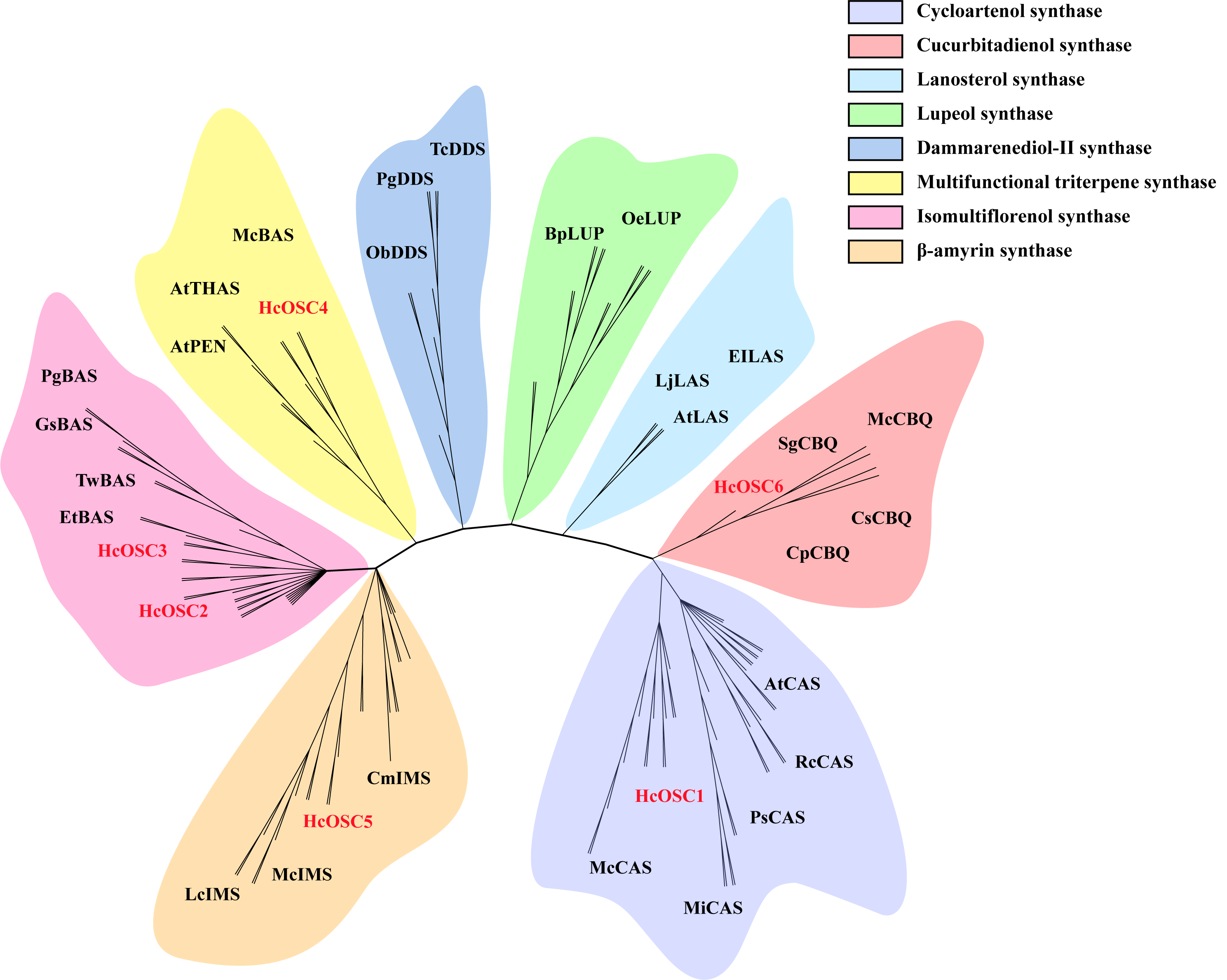
**

**Supplementary Figure 7.** Phylogenetic analysis of the OSCs family of HcOSC1-6 in *Hemelysa chinensis*.

**
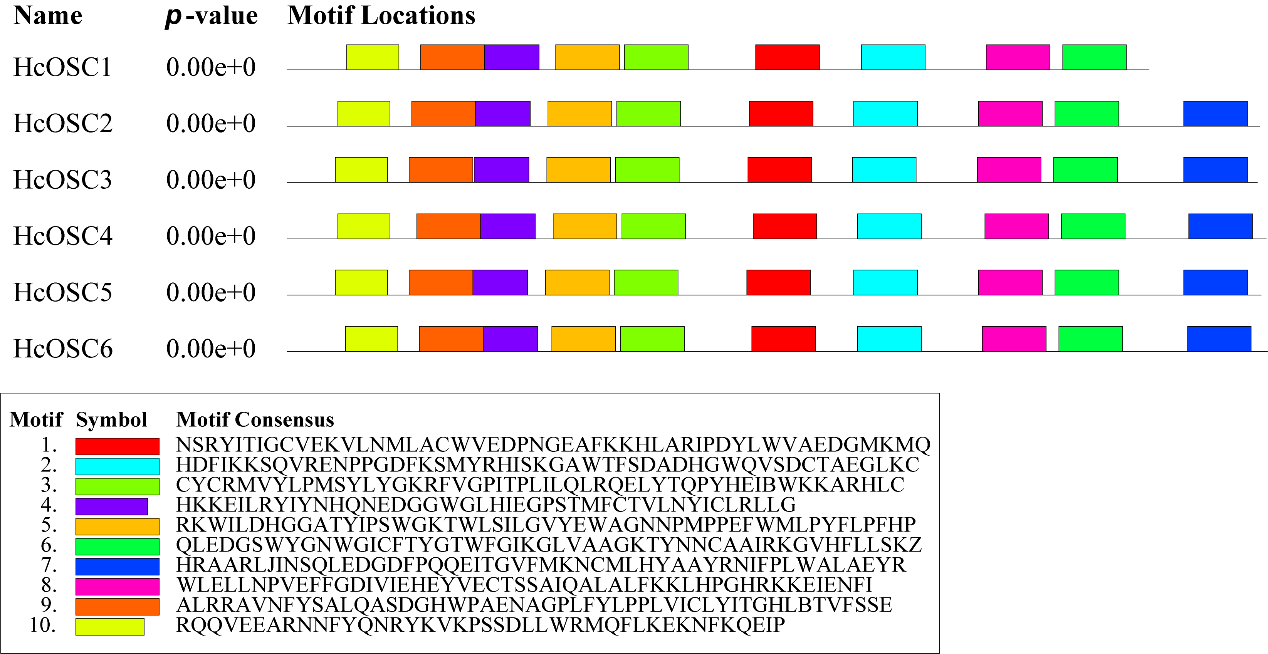
**

**Supplementary Figure 8.** Motif distribution analysis of HCOSC1-6. The 10 color squares in the figure represent the 10 conserved motifs of HcOSC1-6, and each color represents a different amino acid conserved motif each.


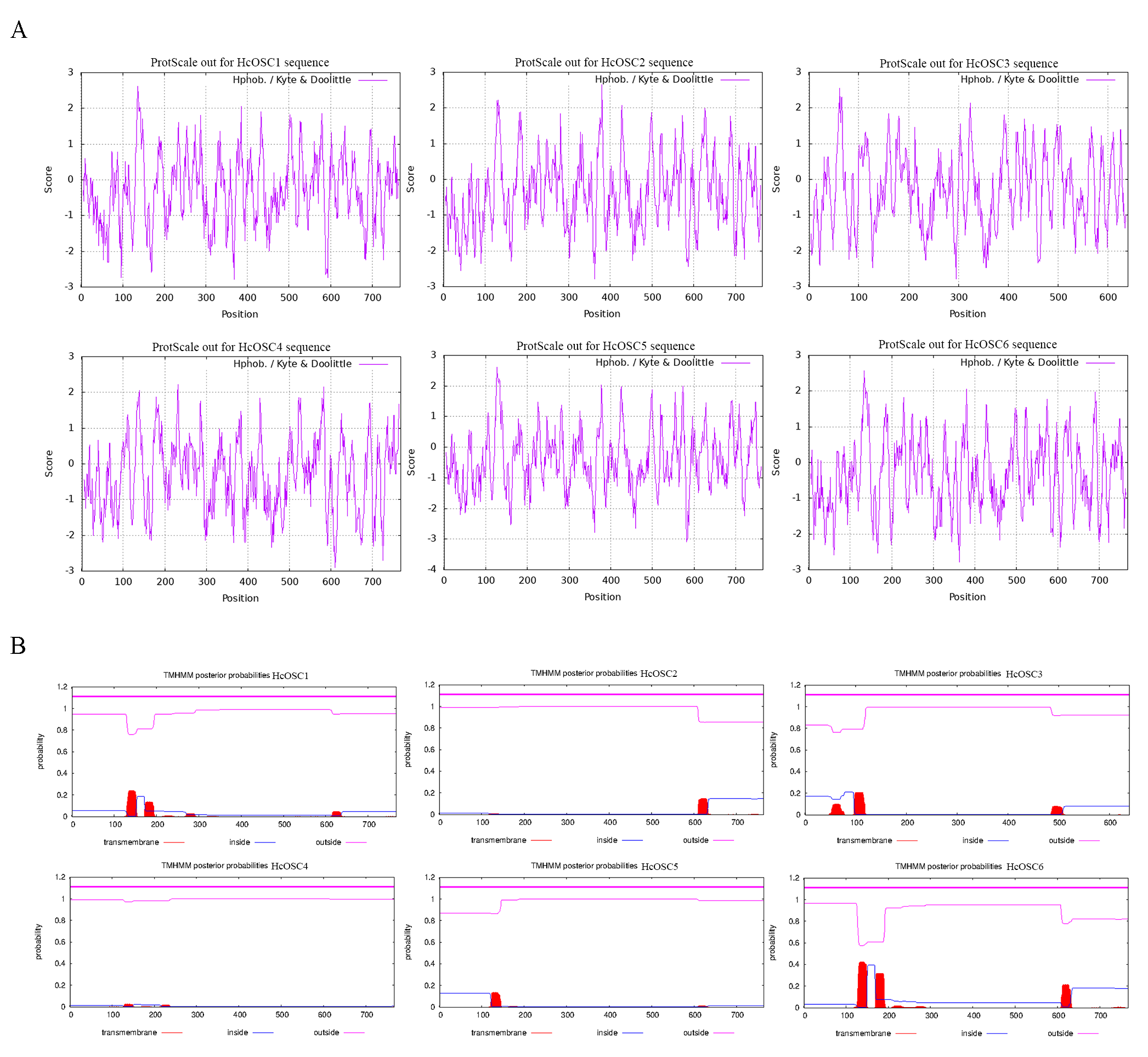


**Supplementary Figure 9.** Hydrophilicity analysis and transmembrane domain predictions of HCOSC1-6.

(A) Hydrophilicity analysis of HCOSC1-6. Protein HcOSC1-6 all indicate hydrophilic.

(B)Transmembrane domain analysis of HCOSC1-6. HcOSC1-3,5,6 is a protein containing a transmembrane region HcOSC4 does not contain a transmembrane structure.

**Supplementary Table 1.** The primers list of site-specific mutagenesis

| Gene ID | With homology arm primer (on vector pYES2) |
| --- | --- |
| HcOSC6 | 5'F： ttggtaccgagctcggatccATGTGGAAGTTAAAGATAGGAGGAG |
|  | 3'R： cactggcggccgttactagtTCAGAATAAAGCGGCCGG |
| Gene ID | The primers for site-specific mutagenesis |
| P244A | 5'F：TGGTCTGGCAACAATCCTCTTGCACCTGAATTTTGG |
|  | 3'R：TCCAAAATTCAGGTGCAAGAGGATTGTTGCCAGACC |
| E246A | 5'F：AACAATCCTCTTCCACCTGCATTTTGGATATTACC |
|  | 3'R：AAGGTAATATCCAAAATGCAGGTGGAAGAGGATTG |
| F247A | 5'F：ACAATCCTCTTCCACCTGAAGCATGGATATTACC |
|  | 3'R：AGGTAATATCCATGCTTCAGGTGGAAGAGGATTG |
| W248A | 5'F：TCCACCTGAATTTGCAATATTACCTTACTTCCTACC |
|  | 3'R：AGGTAGGAAGTAAGGTAATATTGCAAATTCAGGTGG |
| L250A | 5'F：TCCACCTGAATTTTGGATAGCACCTTACTTCCTACC |
|  | 3'R：AGGTAGGAAGTAAGGTGCTATCCAAAATTCAGGTGG |
| F256A | 5'F：ACTTCCTACCTGCACATCCAGGAAGAATGTGG |
|  | 3'R：ACCACATTCTTCCTGGATGTGCAGGTAGGAAG |
| M261A | 5'F：ATCCAGGAAGAGCATGGTGTCATTGCCGAATGG |
|  | 3'R：ACCATTCGGCAATGACACCATGCTCTTCCTGGATG |
| Y269A | 5'F：TCATTGCCGAATGGTGGCACTACCAATGTCTTAC |
|  | 3'R：AGTAAGACATTGGTAGTGCCACCATTCGGCAATG |
| S273A | 5'F：TGCCGAATGGTGTATCTACCAATGGCATACTTATATGG |
|  | 3'R：TCCATATAAGTATGCCATTGGTAGATACACCATTCGGC |
| L328A | 5'F：ATGCAAGACATTGCATGGGGGTCTATACACCAC |
|  | 3'R：CGTGGTGTATAGACCCCCATGCAATGTCTTGCA |
| W329A | 5'F：ATGCAAGACATTCTGGCAGGGTCTATACACCAC |
|  | 3'R：CGTGGTGTATAGACCCTGCCAGAATGTCTTGCA |
| I332A | 5'F：ACATTCTGTGGGGGTCTGCACACCACGTGTATG |
|  | 3'R：TCATACACGTGGTGTGCAGACCCCCACAGAATG |
| Y336A | 5'F：TCTATACACCACGTGGCAGAGCCCTTCTTTAC |
|  | 3'R：AGTAAAGAAGGGCTCTGCCACGTGGTGTATAG |
| I551A | 5'F：TGGTTGGAGTTGGCAAACCCTGCAGAAACG |
|  | 3'R：ACGTTTCTGCAGGGTTTGCCAACTCCAACC |
| P553A | 5'F：TGGTTGGAGTTGGCAAACCCTGCAGAAACG |
|  | 3'R：ACCAAACGTTTCTGCTGCGTTGATCAACTCC |
| D490E | 5'F：ATGGCTGATCTCTGAGTGTACAGCAGAGGG |
|  | 3'R：ATCCCTCTGCTGTACACTCAGAGATCAGCC |
| C491A | 5'F：ATGGCTGATCTCTGACGCAACAGCAGAGGG |
|  | 3'R：ATCCCTCTGCTGTTGCGTCAGAGATCAGCC |

**Supplementary Table 2.** Physicochemical properties of the nucleotide sequence of HcOSC1-6.

| **No.** | **Number of amino acids** | **Molecular weight** | | **Theoretical pI** |
| --- | --- | --- | --- | --- |
| HcOSC1 | 677 | 77184 | 5.85 | |
| HcOSC2 | 764 | 88149.85 | 6.07 | |
| HcOSC3 | 762 | 87340.60 | 5.94 | |
| HcOSC4 | 769 | 88091.96 | 6.32 | |
| HcOSC5 | 765 | 88165.09 | 6.68 | |
| HcOSC6 | 770 | 88062.21 | 6.34 | |
